# Comparative Proteomic Analysis Reveals the Cross-Talk between the Responses Induced by H_2_O_2_ and by Long-Term *Rice Black-Streaked Dwarf Virus* Infection in Rice

**DOI:** 10.1371/journal.pone.0081640

**Published:** 2013-11-27

**Authors:** Qiufang Xu, Haiping Ni, Qingqing Chen, Feng Sun, Tong Zhou, Ying Lan, Yijun Zhou

**Affiliations:** Institute of Plant Protection, Jiangsu Academy of Agricultural Sciences, Jiangsu Technical Service Center of Diagnosis and Detection for Plant Virus Diseases, Nanjing, P. R. China; University of South Florida College of Medicine, United States of America

## Abstract

Hydrogen peroxide (H_2_O_2_) could be produced during the plant-virus compatible interaction. However, the cell responses regulated by the enhanced H_2_O_2_ in virus infected plant are largely unknown. To make clear the influence of Rice black-streaked dwarf virus (RBSDV) infection on H_2_O_2_ accumulation, we measured the content of H_2_O_2_ and found the H_2_O_2_ level was increased in rice seedlings inoculated with RBSDV. To reveal the responses initiated by the enhanced H_2_O_2_ during plant-virus interaction, the present study investigated the global proteome changes of rice under long-term RBSDV infection. Approximately 1800 protein spots were detected on two-dimensional electrophoresis (2-DE) gels. Among them, 72 spots were found differently expressed, of which 69 spots were successfully identified by MALDI-TOF/TOF-MS. Furthermore, the differentially expressed proteins induced by RBSDV infection were compared to that induced by H_2_O_2_. 19 proteins corresponding to 37 spots, which were differentially expressed under RBSDV infection, were observed differentially expressed under H_2_O_2_ stress as well. These overlapping responsive proteins are mainly related to photosynthesis, redox homeostasis, metabolism, energy pathway, and cell wall modification. The increased H_2_O_2_ in RBSDV infected plant may produce an oxidative stress, impair photosynthesis, disturb the metabolism, and eventually result in abnormal growth. The data provide a new understanding of the pivotal role of H_2_O_2_ in rice-RBSDV compatible interaction.

## Introduction

Hydrogen peroxide (H_2_O_2_) is one of the most important types of reactive oxygen species (ROS) and has attracted much attention during the last decades. It is well known that ROS plays a pivotal role in the defense response of plants against pathogen [1,2]. The accumulation of H_2_O_2_ during the plant- pathogen incompatible interaction was correlated with the establishment of disease resistance [3,4]. H_2_O_2_ can act as a local signal for hypersensitive response as well as a diffusible signal for the induction of defensive genes in adjacent cells [5]. Previous studies demonstrated that H_2_O_2_ has dual functions in plant. As a signaling molecule, it has been proved to modulate gene expression and participate in various processes, such as cell growth, pathogen defense, programmed cell death, hormonal responses, photosynthesis regulation, and signal transduction [6-8]. On the other hand, H_2_O_2_ is highly reactive and toxic. The steady-state level of H_2_O_2_ should be tightly controlled in plant. Excessive production of H_2_O_2_ can alter the redox state of the cells, damage a large variety of subcellular constitutions such as proteins and nucleic acids, and lead to oxidative destruction of cells [9]. In the compatible interaction, the production of H_2_O_2_ was considered to be a non-specific response and the function of H_2_O_2_ produced during pathogen infection was rarely studied.

In plant-virus incompatible interaction, the generation of H_2_O_2_ is associated with resistance to virus. Extraneous low concentrations of H_2_O_2_ in tobacco could suppress necroses caused by *Tobacco mosaic virus* [10]. Resistant rice variety inoculated with *Rice stripe virus* resulted in an increase of H_2_O_2_ [11]. However, a rapid accumulation of H_2_O_2_ and an imbalance in the antioxidative systems had also been observed in plant-virus compatible interaction [12]. The H_2_O_2_ level was found increased in Plum pox virus infected pea leaves; this level is enhanced during the development of the disease and is accompanied with an imbalance in the antioxidative systems [13]. Moreover, the increase of H_2_O_2_ is more remarkable in the virus susceptible plant than in the resistant plant [14]. Nevertheless, relatively little information is known about the involvement of H_2_O_2_ in symptom development and pathogenesis in plant-virus compatible interactions.

RBSDV, a member of the genus *Fijivirus* in the family *Reoviridae*, could infect rice and maize, and leads to rice black streaked dwarf disease and maize rough dwarf diseases, respectively [15,16]. Recently, the diseases caused by RBSDV have proliferated rapidly and have become economically destructive diseases in China [17]. The virus is mainly transmitted by *Laodelphax striatellus* Fallen, the small brown planthopper (SBPH), in a persistent and circulative manner [18,19]. The plants infected with RBSDV are characterized by inhibited plant growth, darkened leaves, white tumors or black streaked swellings along the veins on the back of leaf blades and stems [20]. Proteomic and microarray investigations revealed that ascorbate peroxidases (APX) and catalases (CAT) were up-regulated undergoing long-term RBSDV infection in maize plant [21,22]. CAT and APX are major H_2_O_2_-scavenging enzymes and are believed to be crucial in determining the steady-state level of H_2_O_2_ [8]. The upregulation of these proteins indicates that H_2_O_2_-scavenging pathway is active in plant cells to maintain the H_2_O_2_ balance. Interestingly, the viral protein p5b encoded by RBSDV genomic segment S5 could interact with CAT and APX in rice plant [23], and the protein encoded by segment S6 could interact with thylakoid-bound ascorbate peroxidase [24]. The biological significance of the interactions is still unknown. A stimulating hypothesis is that the interactions between RBSDV and the H_2_O_2_-scavenging enzymes inhibit or increase the H_2_O_2_ scavenging, and cause an imbalance of H_2_O_2_. 

In this study, to confirm the hypothesis, the content of H_2_O_2_ was measured, and the increase of H_2_O_2_ was observed after long-term RBSDV infection. Previous work had revealed the protein network elicited by H_2_O_2_ in rice by a proteomic method [25]. In order to investigate the cell processes the H_2_O_2_ involved in during systemic virus infections, a proteomic approach was applied to analyze the global cellular response to RBSDV infection in rice, and the changes in protein expression were compared to that induced by H_2_O_2_ stress at proteome level. A series of responsive proteins were found regulated by both RBSDV infection and H_2_O_2_ stress. The results presented in this study provide the framework for further functional studies of H_2_O_2_ produced under long-term virus infection.

## Materials and Methods

### Plant materials, virus inoculation and plant growth conditions

The susceptible rice cultivar Huai 5 (*Oryza sativa japonica*) was used for virus inoculation. Rice seeds were submerged in water inside a container for two days, and then sown in a 1 L beaker and kept in a greenhouse at 25°C with a light/dark cycle of 16/8 h. Nine-day-old rice seedlings were exposed to viruliferous SBPHs (approximately three viruliferous insects per seedling) in each of the inoculation chamber for 3 days. The seedlings that were mock-inoculated with the virus-free SBPH were used as controls. After the insects were removed from the plants by gentle brushing, the inoculated seedlings were transplanted to an insect-free glasshouse at 25 ± 3 °C under natural sunlight. The aerial parts of the whole plants were collected 50 days post-inoculation (dpi), a time when the dwarf symptom could be significantly observed in virus-inoculated seedlings. 

To verify that the dwarf symptoms were caused by RBSDV infection, RBSDV genome segments S9-1 and S10 was examined by RT-PCR analysis [26,27]. PCR was performed with the virus specific primers listed in Table S1. The expected sizes of S9-1 and S10 were 1044 bp and 1677 bp, respectively. The amplified fragments of the expected size were confirmed by electrophoresis in 1% (w/v) agarose gels.

### Protein samples preparation

Proteome analysis was performed in three biological repeats. The aerial part of the rice seedlings were used for protein extraction. Proteins were extracted using the TCA/acetone precipitation method according to Damerval et al. with several modifications [28]. One gram of rice was ground to a fine powder in liquid nitrogen, and the powder was later precipitated by 30 ml of cold acetone (-20°C) with 10% w/v TCA and 0.07% β-mercaptoethanol at -20 °C overnight. After centrifugation at 13,000 g at 4 °C for 30 min, the pellets were washed three times with 30 ml of ice-cold acetone containing 0.07% β-mercaptoethanol and lyophilized. The resulting powder was dissolved in lysis buffer (7 M urea, 2 M thiourea, 4% w/v CHAPS, 65 mM DTT and 2% IPG buffer (pH 3-10)). After incubation at 30 °C for 1 h, the suspension was centrifuged at 40,000 g at 4 °C for 30 min to remove the insoluble debris. The protein concentration was quantified using a 2-D Quant kit (GE Healthcare). 

### 2-DE, gel staining and image analysis

The 2-DE was performed as described by Xu et al. [29] with some modification. The IPG strips (24 cm, pH 4-7 nonlinear, GE Healthcare) were passively rehydrated at 25 °C for 12 h with 450 μl of rehydration buffer (7 M urea, 2 M thiourea, 2% CHAPS, 30 mM DTT, 0.5% IPG buffer (pH 4-7) and 0.004% bromophenol blue) containing 800 μg of protein. Isoelectric focusing was performed on an Ettan IPGphor II (GE Healthcare) at 20 °C, under the following conditions: 100V for 1 h, 250 V for 1 h, 500V for 1 h, 1000V for 1 h, 8000 V for 3 h, and 8000 V with a total of 68000 vhs. After focusing, the strips were incubated in 10 ml of equilibration buffer (6 M urea, 2% w/v SDS, 75 mM Tris-HCl at pH 8.8, 30% v/v glycerol, 0.002% w/v bromophenol blue, and 1% w/v DTT) for 15 min and subsequently in equilibration buffer containing 2.5% w/v iodoacetamide instead of 1% DTT for another 15 min. For the second dimension electrophoresis, the proteins were separated on 12.5% vertical polyacrylamide gels using an Ettan Dalt six electrophoresis system (GE Healthcare). The electrophoresis was performed at 2 watts/gel for 45 min followed by 17 watts/gel until the dye front reached approximately 1cm from the bottom of the gels.

2-DE gels were stained by a modified colloidal Coomassie G-250 staining [30] and scanned by UMAX PowerLook 2100XL scanner (UMAX Systerms, Germany). A total of six gels (three of the best-matched replicate gels from three independent experiments for each treatment) were analyzed using ImageMaster 2D Platinum software version 7.0 (GE Healthcare). The images were analyzed according to the manufacturer’s instructions. Spot detection and gel matching were performed automatically. Protein expression was quantified using the normalized percentage volumes (vol. %), a ratio of the volume of a particular spot to the total volume of all of the spots present on a gel. The match analysis was manually edited to correct the mismatched spots. To calculate the significant differences, the expression levels of the mock- and RBSDV- inoculation groups were analyzed using Student’s t-test. The protein spots with vol. % ≥1.5 and P ≤ 0.05 were defined as differentially expressed proteins (DEPs).

### In-gel digestion, MALDI-TOF/TOF-MS and data analysis

The DEPs were manually excised from their gels for proteolytic digestion [29]. The excised gel pieces were destained with 200 μl of 100 mM ammonium bicarbonate (NH_4_HCO_3_) in 30% acetonitrile. After removing the destaining buffer, the gel pieces were dried in a SpeedVac and rehydrated with 30 μl of 50 mM NH_4_HCO_3_ containing 50 ng trypsin (sequencing grade; Promega, Madison, WI, USA). The peptides were extracted three times with 0.1% trifluoroacetic acid (TFA) in 60% acetonitrile after an overnight digestion at 37 °C. The supernatants were pooled together and lyophilized, and the resulting peptides were dissolved in 50% acetonitrile containing 0.5% TFA and kept at -80 °C until use in mass spectrometry. A protein-free gel piece was used as a control to identify the autoproteolysis products derived from trypsin.

MALDI-TOF/TOF-MS was conducted with a 4800 MALDI TOF/TOF™ Analyzer (Applied Biosystems, Foster City, CA, USA) according to Meisrimler et al. [31]. The spectra of the proteins were searched against the NCBI nonredundant protein (NCBInr) database with the taxonomy *Oryza sativa* 2010.12.10 using MASCOT 2.1 (MatrixScience, London, UK). The spectra that were not identifiable were researched in the NCBInr database with the taxonomy of all entries. The search parameters were set as follows: enzyme: trypsin; fix modifications: carbamidomethyl (C); mass values: monoisotopic; peptide mass tolerance: ± 100 ppm; fragment mass tolerance: ± 0.8 Da; peptide charge state: 1+; max missed cleavages: 1. Proteins identified by MALDI-TOF/TOF-MS with C.I. % scores above 95% were deemed significant.

### Measurements of photosynthesis and H_2_O_2_ level

The net photosynthetic rates, stomatal conductance, intercellular CO_2_ concentrations and transpiration speeds of the third and fourth rice leaves of each sample were measured using a LI-6400 XT portable photosynthesis system with a Red/Blue LED light source (LI-COR Inc., Lincoln, NE, USA). At least ten leaves were measured for each sample. 

The H_2_O_2_ accumulation in mock- or virus- inoculated leaves was measured according to Wan et al. [25]. One gram of rice seedling leaves were ground with a pestle and mortar in liquid nitrogen to a fine powder, and suspended in 10 ml of 5 mM titanium sulfate. After incubation for 1 h at room temperature, debris was discarded by centrifugation at 12,000 g for 10 min at 25°C. The oxidation of titanium sulfate was recorded by reading the *A*410 using a PerkinElmer Lambda 25 UV/VIS spectrometer. The H_2_O_2_ concentrations were calculated according to a standard calibration plot, and expressed as μmol H_2_O_2_ g^-1^ FW.

### Quantitative real-time PCR

Quantitative real-time PCR was performed to quantify the transcriptional levels of genes corresponding to the DEPs. The sequences of the identified proteins were searched against the NCBI database using tBLASTn. The corresponding gene sequences were selected for primer design, and the primers designed using primer express 3.0 software were shown in Table S1. Total RNA was extracted with Trizol (Invitrogen) and treated with DNase I (Promega, WI, USA) to eliminate the DNA. The integrity of the RNA samples was assessed by agarose gel electrophoresis, and RNA concentrations were measured using a spectrophotometer. Reverse transcription was performed using 1 μg of RNA. The cDNA was synthesized using an M-MLV RTase cDNA synthesis kit (TaKaRa). The iQ5 Real-Time PCR System (Bio-Rad) was used to perform RT-qPCR. The rice UBQ5 gene was used as internal standard, and experiments were performed in triplicate. Amplification was performed in a 20 μl reaction mixture containing 1μl of cDNA, 1 μl of forward and reverse primers, 8 μl of ddH_2_O and 10 μl of SsoFast^TM^ EvaGreen supermix (Bio-Rad). After an initial denaturation at 95 °C for 20 s, 40 cycles of amplification were carried out: denaturation at 95 °C for 10 s, annealing at 58 °C for 15 s and extension at 72 °C for 15 s. Melting curves were obtained and quantitation of the data was performed using the iQ5 software (Bio-Rad) with the relative quantification (ddCt) model.

## Results

### RBSDV infection elevated the H_2_O_2_ level in rice

**Figure 1:**
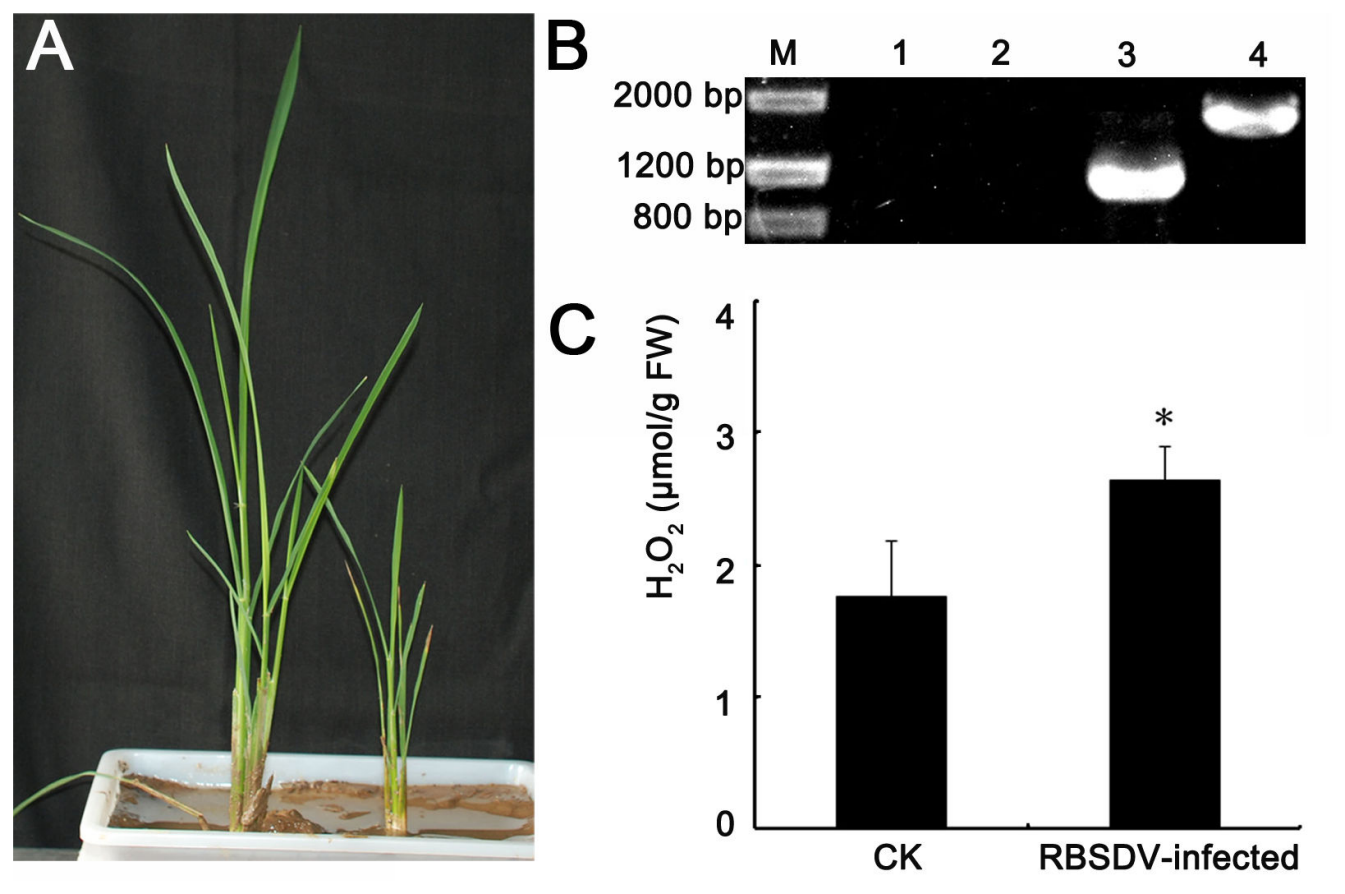
Long-term RBSDV infection resulted in an increase of H_2_O_2_ in rice. (A) An RBSDV - infected rice plant (right) is compared to the mock-inoculated plant (left) at 50 dpi. (B) RT-PCR analysis of the RBSDV genome segments S9-1 and S10 in mock-inoculated rice (lanes 1 and 2) and in RBSDV-inoculated rice (lanes 3 and 4). (C) The accumulation of endogenous H_2_O_2_ in rice.

The rice seedlings infected with RBSDV exhibited notable stunting symptoms (Figure 1A). The height and width of the infected plants were only 46.2% and 56.7% of that of the control. The virus genome segments S9-1 and S10 could be detected by RT-PCR in the virus-infected rice leaves but not in the mock-infected plants (Figure 1B), indicating that the virus successfully infected the rice plant and consequently led to the dwarf. To verify whether the level of H_2_O_2_ was changed by RBSDV-infection in rice, the content of H_2_O_2_ was measured. An increasing H_2_O_2_ was found in RBSDV infected rice plant. The H_2_O_2_ concentration in virus infected plants was 2.64 μmol g^-1^ FW whereas it was 1.75 μmol g^-1^ FW in the control. As shown in Figure 1C, the H_2_O_2_ content in RBSDV infected plants was 1.5 times as much as the control. 

### Proteomic analysis of rice plants in response to RBSDV infection

**Figure 2:**
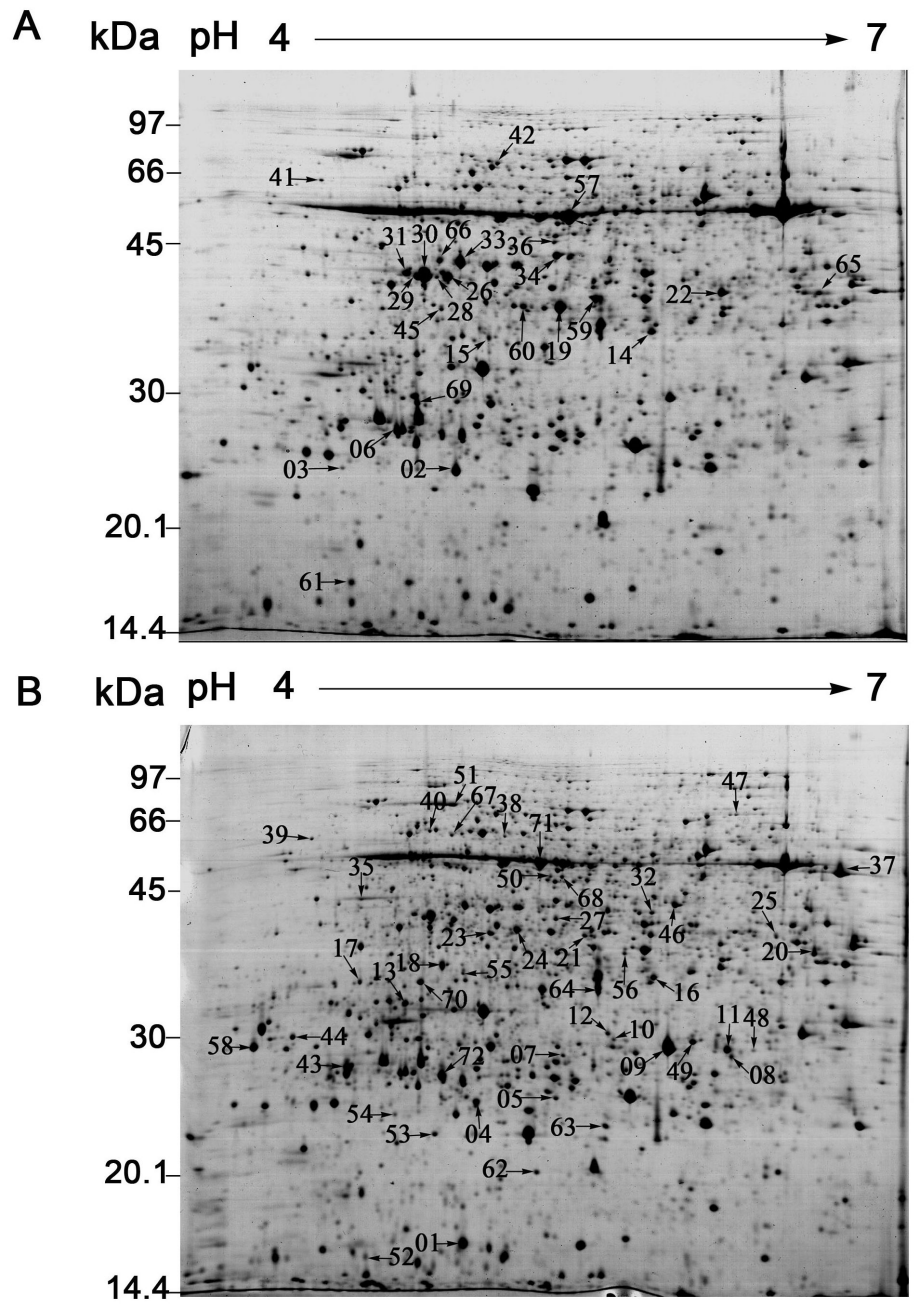
The mock- and RBSDV-infected rice proteomes display 72 differentially expressed protein spots. The Proteins (800 μg) were separated on 24 cm pI 4-7 non-linear gradient IPG strips and with 12.5% SDS-PAGE. The gels were stained with CBB G-250 according to the blue silver method. A total of 72 proteins were differentially expressed in response to RBSDV infection. The down-regulated proteins were labeled in the 2-DE gel image of the mock-infected rice (A), and the up-regulated proteins were marked in the gel of the RBSDV-infected rice (B).

To reveal the changes in protein expression under RBSDV stress in rice, the proteome profiles of mock- and virus-infected plants were analyzed by 2-DE. Approximately 1800 detectable spots were visualized on each gel by Coomassie Brilliant Blue staining (Figure 2). Three independent experiments were conducted to ensure that the protein abundance changes were reproducible and significant. 72 spots that showed at least a 1.5-fold increase or decrease in abundance (P≤0.05) were considered to change significantly between the mock- and virus-infected plants. Of these spots, 25 spots were down-regulated (marked in Figure 2A) and 47 spots were up-regulated (marked in Figure 2B). 

**Figure 3:**
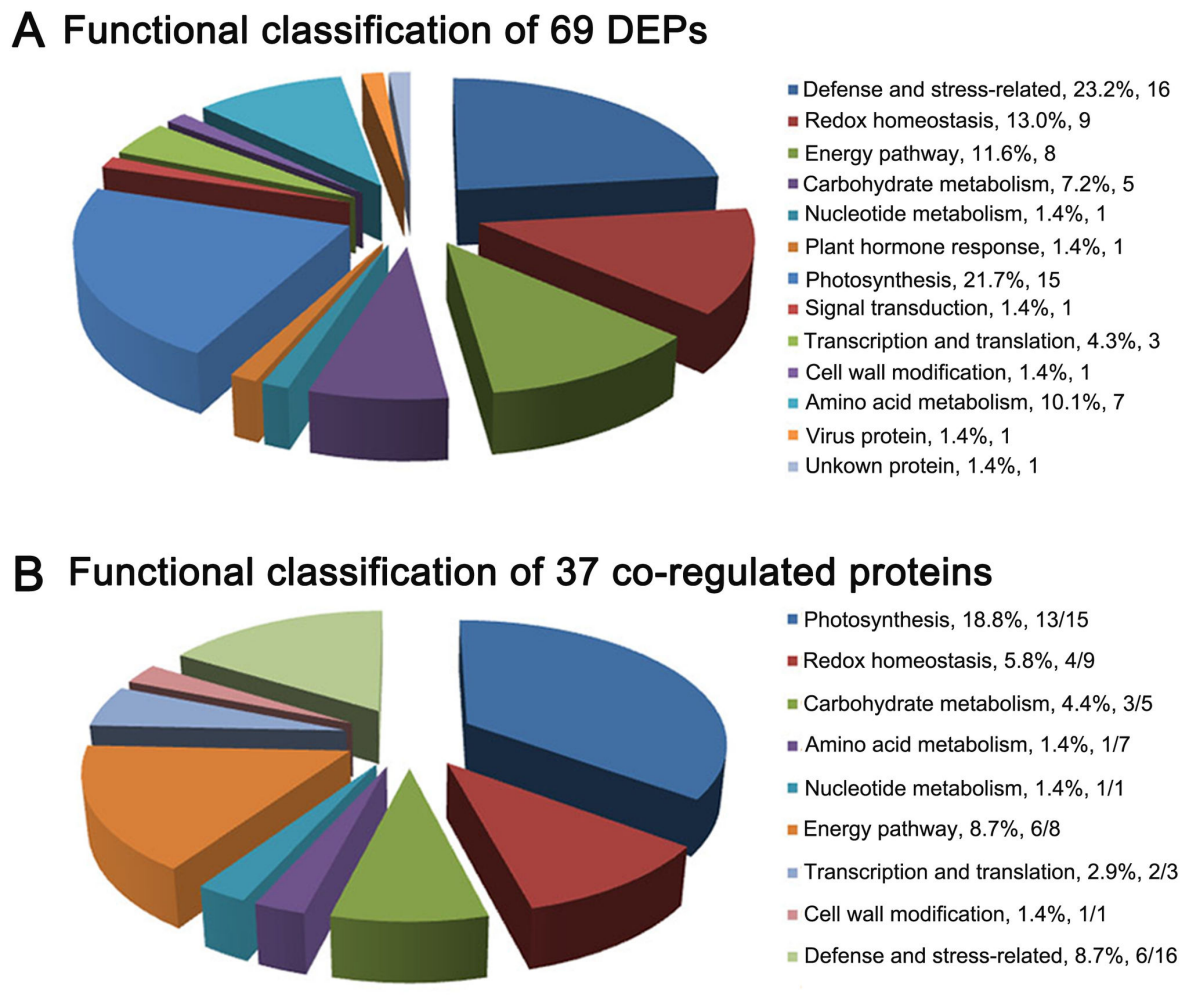
Classification of the differentially expressed proteins (DEPs). A, functional classification of the 69 DEPs in RBSDV-infected rice plant. Function, (number of DEP spots in each functional classification/ total DEP spots) % and the number of DEP spots in each classification were shown. B, functional classification of the 37 DEP spots co-regulated by RBSDV infection and by H_2_O_2_ stress. Function, (number of co-regulated protein spots in each functional classification/total DEP spots) % and the number of the co-regulated protein spots/total DEP spots in each classification were shown.

To identify the proteins that were differentially expressed due to the RBSDV infection, all 72 spots with a threshold greater than 1.5-fold were excised and analyzed by MALDI-TOF/TOF-MS. 69 spots were successfully identified. 43 of these spots have been deposited in the current database as putative functional proteins. The remainder of the spots was without specific function in the database and was annotated using the Uniprot Knowledgebase (www.uniprot.org) or the NCBI (www.ncbi.nlm.nih.gov) database with BLASTP. The results of the identification of the 69 spots were shown in Table 1. The corresponding homologues with annotations were listed in Table S2 and the peptide sequences identified by MOLDI-TOF/TOF-MS were shown in Table S3. According to the annotations, the identified proteins were involved in the following 12 categories according to their functional features, including defense and stress related proteins, photosynthesis, redox homeostasis, energy pathway, amino acid metabolism, carbohydrate metabolism, nucleotide metabolism, transcription and translation, cell wall modifications, plant hormone responses, signal transduction and virus proteins (Table 1, Figure 3A). About 80% different proteins were related to defense and stress response, photosynthesis, redox homeostasis, energy pathway and amino acid metabolism. The virus infection seems to have triggered the defense and stress response, and impaired the photosynthesis, as most of the DEPs related to defense and stress response were found up-regulated, and most of those proteins related to photosynthesis were down-regulated (Table 1).

**Table 1:** The 69 differentially expressed proteins identified in rice under long-term RBSDV infection.

**Spot no.**	**Protein name**	**^a^Accession no**	**^b^TMW /EMW (KDa)**	**^c^ Tp*I* / Ep*I***	**^d^Peptides**	**Protein score**	**Protein score C.I.%^e^**	**Best ion score**	**Average fold change (RBSDV /CK)**	**Anova**
**Defense and stress related proteins**										
01	Thaumatin-like protein	gi|115489688	18.5 /17.8	5.07 /5.23	3	366	100	154	2.03	7.02E-04
08	Putative chitinase	gi|54291729	32.7 /284	6.08 /6.51	12	777	100	118	2.15	1.60E-04
09	Putative chitinase	gi|54291729	32.7 /29.1	6.08 /6.29	12	605	100	110	3.87	1.19E-04
10	Putative chitinase	gi|54291729	32.7 /30.6	6.08 /6.05	8	266	100	60	2.99	3.27E-03
11	Putative chitinase	gi|54291729	32.7 /28.8	6.08 /6.50	12	711	100	114	3.30	3.07E-04
12	Putative chitinase	gi|54291729	32.7 /30.8	6.08 /6.01	13	627	100	98	5.55	2.98E-04
44	Putative class III chitinase	gi|125531926	31.2 /30.3	4.48 /4.45	8	479	100	97	2.99	7.08E-05
17	Beta-1,3-glucanase precursor	gi|4097942	34.8 /35.2	4.72 /4.60	5	216	100	94	2.10	2.04E-04
38	Endo-1,3-beta-glucanase	gi|115442217	67.7 /62.3	5.32 /5.39	17	354	100	88	2.89	3.83E-03
39	Chloroplast heat shock protein 70	gi|115463081	48.6 /61.8	4.57 /4.49	10	413	100	130	2.58	4.38E-04
51	Heat shock cognate 70 kDa protein	gi|108864707	67.6 /75.9	4.97 /5.10	24	825	100	123	9.15	6.31E-03
53	Ribonuclease 3 precursor	gi|149392262	28.5 /23.7	5.57 /4.98	14	768	100	132	RBSDV only	8.54E-05
54	Ribonuclease 3 precursor	gi|149392262	28.5 /24.8	5.57 /4.68	12	530	100	110	RBSDV only	8.47E-04
62	Salt stress root protein RS1	gi|115435500	21.8 /22.3	4.92 /5.62	8	330	100	110	1.84	1.04E-03
69	Salt stress root protein RS1	gi|115435500	21.8 /29.6	4.92 /4.85	10	291	100	122	-1.72	2.26E-03
48	r40c1 protein	gi|24899397	42.2 /28.8	6.25 /6.58	11	327	100	99	3.05	3.13E-03
**Photosynthesis**										
02	Chlorophyll a/b binding protein	gi|125555124	26.4 /24.6	5.75 /5.14	5	165	100	93	-2.22	6.02E-05
06	Chlorophyll a/b binding protein	gi|108864186	24.0 /26.1	4.73 /4.69	11	693	100	118	-2.16	1.46E-04
37	Ribulose-1,5-bisphosphate carboxylase/oxygenase large subunit	gi|11466795	53.4 /51.8	6.22 /6.85	26	796	100	208	2.22	1.00E-03
63	Ribulose-1,5-bisphosphate carboxylase/oxygenase large subunit	gi|109156602	54.3 /24.4	6.33 /6.02	11	435	100	118	1.67	6.40E-04
19	Fructose-bisphosphate aldolase	gi|108864048	41.8 /39.5	6.07 /5.66	22	945	100	153	-6.93	1.12E-05
49	Fructose-bisphosphate aldolase	gi|115463789	36.6 /30.3	6.56 /6.40	18	1140	100	162	2.55	9.52E-05
60	Fructose-bisphosphate aldolase	gi|108864048	41.8 /39.8	6.07 /5.56	8	298	100	131	-2.39	1.79E-03
29	RuBisCO activase small isoform precursor	gi|8918361	48.1 /43.1	5.85 /4.89	26	756	100	127	-2.16	5.08E-06
30	RuBisCO activase small isoform precursor	gi|62733297	52.8 /43.7	5.59 /4.94	20	530	100	102	-2.11	1.45E-05
31	RuBisCO activase small isoform precursor	gi|8918361	48.1 /43.9	5.85 /4.79	25	974	100	165	-2.41	4.53E-05
28	RuBisCO activase small isoform precursor	gi|8918361	48.1 /42.6	5.85 /5.04	23	856	100	130	-2.14	1.53E-04
33	Phosphoglycerate kinase	gi|125552851	30.5 /44.8	6.86 /5.20	17	1030	100	145	-1.90	9.25E-03
66	Phosphoglycerate kinase	gi|125552851	30.5 /44.3	6.86 /5.05	17	1080	100	119	-1.90	2.91E-04
26	Phosphoribulokinase	gi|115448091	45.2 /42.3	5.68 /5.12	21	1400	100	186	-2.89	2.85E-05
65	Glyceraldehyde-3-phosphate dehydrogenase A	gi|115458768	43.0 /40.5	7.62 /6.74	22	972	100	155	-1.99	3.33E-02
**Energy pathway**										
24	ATP synthase CF1 beta subunit	gi|50233978	54.0 /41.5	5.38 /5.46	30	1490	100	146	2.13	8.56E-04
25	ATP synthase CF1 beta subunit	gi|50233978	54.0 /40.1	5.38 /6.65	15	392	100	93	2.59	4.51E-04
57	ATP synthase CF1 beta subunit	gi|50233978	54.0 /53.7	5.38 /5.57	29	1650	100	182	-3.45	2.19E-04
68	ATP synthase CF1 beta subunit	gi|50233978	54.0 /50.2	5.38 /5.68	20	971	100	113	1.73	2.05E-04
71	ATP synthase CF1 beta subunit	gi|50233978	54.0 /54.5	5.38 /5.58	30	1440	100	178	2.14	1.89E-03
23	ATP synthase beta subunit	gi|56784991	45.9 /41.2	5.33 /5.35	18	1070	100	149	2.21	9.53E-04
22	ATP synthase gamma chain	gi|115472339	40.1 /41.2	8.60 /6.48	15	482	100	80	-3.01	6.69E-05
72	ATP synthase gamma chain	gi|115472339	40.1 /26.1	8.60 /5.03	15	482	100	80	1.80	3.09E-02
**Redox homeostasis**										
04	L-ascorbate peroxodase 1, cytosolic	gi|158512874	27.2 /25.2	5.31 /5.29	11	378	100	147	2.27	1.28E-04
05	Glutathione transferase GST 23	gi|115479659	25.3 /25.8	5.50 /5.66	14	528	100	97	2.26	8.68E-04
21	Catalase	gi|283050393	57.1 /41.5	6.60 /5.84	17	924	100	102	2.22	1.02E-03
64	Peroxidase	gi|20286	33.3 /34.8	5.77 /5.94	7	616	100	195	1.86	1.24E-03
15	Class III peroxidase 29 precursor	gi|115445243	34.8 /35.2	5.32 /5.35	10	639	100	145	-2.43	9.01E-05
61	Thioredoxin peroxidase	gi|115444771	23.3 /16.8	6.15 /5.85	12	723	100	131	-1.73	4.71E-03
14	Putative ferredoxin-NADP(H) oxidoreductase	gi|41052915	41.1 /35.6	7.98 /6.18	20	1040	100	147	-1.94	5.72E-06
40	Protein disulfide isomerase	gi|62546209	57.1 /63.4	4.95 /4.92	11	117	100	66	2.44	9.84E-06
67	Protein disulfide isomerase	gi|7209794	33.5 /62.5	4.81 /5.10	17	576	100	124	1.71	2.21E-03
**Carbohydrate metabolism**										
18	Fructokinase 2	gi|115474481	35. 9 /36.5	5.02 /4.96	20	1020	100	180	2.86	2.05E-05
45	Fructokinase 2	gi|115474481	35.9 /39.6	5.02 /5.07	23	897	100	165	-2.43	5.53E-04
32	Enolase	gi|110288667	51.9 /43.8	5.72 /6.19	19	912	100	137	3.04	1.97E-04
36	ADP-glucose pyrophosphorylase small subunit	gi|217075932	54.8 /50.4	6.77 /5.52	20	1150	100	183	-2.17	6.80E-05
55	Pyruvate dehydrogenase E1 component subunit beta	gi|115477529	40.2 /35.9	5.25 /5.21	4	240	100	96	RBSDV only	1.69E-04
**Amino acid metabolism**										
50	Wheat adenosylhomocysteinase-like protein	gi|29367605	53.9 /50.2	5.62 /5.65	7	215	100	105	2.75	4.46E-03
46	5-methyltetrahydropteroyltriglutamate -homocysteine methyltransferase	gi|108862994	66.9 /44.2	5.92 /6.32	19	659	100	162	4.17	6.22E-04
20	5-methyltetrahydropteroyltriglutamate-homocysteine methyltransferase	gi|108862990	79.3 /39.7	7.19 /6.77	14	687	100	187	2.20	1.13E-02
07	Glutamine synthetase	gi|218191527	40.5 /28.5	5.69 /5.59	9	388	100	134	2.10	7.16E-03
42	ATP-dependent zinc metalloprotease FTSH 1	gi|115470052	72.9 /73.8	5.51 /5.38	27	991	100	140	-2.26	1.77E-04
58	Carboxyl-terminal peptidase-like	gi|55296403	45.4 /29.2	5.98 /4.36	4	310	100	112	2.54	9.39E-04
70	4-nitrophenylphosphatase	gi|115459134	39.8 /35.3	6.75 /4.85	17	780	100	147	1.77	3.57E-03
**Nucleotide metabolism**										
59	Putative mRNA binding protein precursor	gi|115471157	41.3 /40.3	7.68 /5.90	15	813	100	118	-2.00	5.74E-04
**Transcription and translation**										
27	Elongation factor 2	gi|115446385	95.0 /43.5	5.85 /5.66	23	1060	100	149	2.25	6.04E-04
16	Translational elongation factor Tu	gi|17225494	50.6 /35.9	6.19 /6.21	16	570	100	150	2.13	5.82E-04
34	Chloroplast translational elongation factor Tu	gi|6525065	50.6 /46.2	6.05 /5.49	24	1400	100	163	-2.08	7.70E-06
**Cell wall modification**										
41	Hydroxyproline-rich glycoprotein-like	gi|115445387	48.4 /62.9	5.05 /4.55	10	205	100	84	-3.16	9.44E-04
**Plant hormone response**										
13	Abscisic stress ripening protein	gi|116309406	25.3 /33.2	4.92 /4.73	12	446	100	115	2.16	9.21E-05
**Signal transduction**										
43	Receptor-like protein kinase DUF26	gi|115461070	27.9 /26.6	5.01 /4.57	12	772	100	115	3.30	2.36E-03
**Viral protein**										
56	P9-1 protein (Rice black streaked dwarf virus)	gi|15387604	40.1 /39.6	5.69 /6.07	7	391	100	96	RBSDV only	1.40E-04
**Unknown protein**										
47	unknown protein	gi|19386746	28.3/ 70.6	8.75 /6.53	2	71	98.87	62	2.93	1.47E-03

a: Accession number of the protein in the NCBInr database;

b: TMW/EMW: molecular mass of predicted protein/of protein on the gel.

c: Tp*I* predicted/EpI: pI of predicted proteins/of proteins on the gel.d: Number of identified Peptides.

e: Protein score C.I.%: Protein score confidence interval percentage.

### The overlapping responsive proteins under H_2_O_2_ stress and RBSDV infection

An H_2_O_2_ stress-responsive protein network in rice seedling had been revealed by Wan et al. [25]. To obtain the overlapping proteomic profile of rice under RBSDV infection and H_2_O_2_ stress, we compared the DEPs identified in this study with the responsive proteomic profile of rice under H_2_O_2_ treatment published previously [25]. Under RBSDV infection, 45 proteins corresponding to 69 identified spots was differentially expressed (Table 1). Of these proteins, 19 proteins corresponding to 37 spots were also differentially expressed under H_2_O_2_ stress. Moreover, in comparison with previously published proteomic and microarray data [21,22], nine of 19 proteins were also detected differentially expressed under RBSDV infection in maize. The expression patterns of these RBSDV and H_2_O_2_ co-regulated proteins were shown in Table 2 and Figure 3B. Among these co-regulated proteins, some proteins had both up- and down- regulated change patterns in abundance. Likewise, many similar phenomena were also observed in other previously reported proteomics studies [32-34]. 

**Table 2:** Proteins differentially expressed under both RBSDV infection and H_2_O_2_ stress.

**Protein names**	**Rice under RBSDV infection (This study)**	**Maize under RBSDV infection [21,22]^a^**	**Rice under H_2_O_2_ stress [25]**
**Photosynthesis**			
Chlorophyll A-B binding protein	↓**^b^**		↓
Glyceraldehyde-3-phosphate dehydrogenase A	↓	↓	↓↑
Ribulose-1,5-bisphosphate carboxylase/oxygenase large unit	↑	↑	↓
Fructose-bisphosphate aldolase	↑↓	↑	↓
Phosphoribulokinase	↓		↑
Rubisco activase small isoform precursor	↓	↑	↓↑
**Redox homeostasis**			
Ascorbate peroxidase	↑	↑	↓
Glutathione S-transferase	↑		↑
Protein disulfide isomerase	↑		↑
**Carbohydrate metabolism**			
Fructokinase-2	↓↑	↑	↓
ADP-glucose pyrophosphorylase	↓	↑	↑
UDP-glucose pyrophosphorylase		↑	↑
**Amino acid metabolism**			
Glutamine synthetase	↑		↑
Cysteine synthase		↓	↑
**Nucleotide metabolism**			
mRNA binding protein precursor	↓		↓↑
**Energy pathway**			
ATP synthase CF1 beta subunit	↓↑	↓↑	↑
ATP synthase beta subunit	↑		↑
**Transcription and translation**			
Translational elongation factor Tu	↓↑	↑	↓↑
Elongation factor P		↓	↑
**Cell wall modification**			
Hydroxyproline-rich glycoprotein-like	↓		↓
**Defense and stress related protein**			
Chitinase	↑		↓
Heat shock cognate 70 kDa protein	↑		↓

^a^ The number of the cited references.

^b^ ↑ and ↓ represent up-regulated and down-regulated expression of proteins.

### Transcriptional profiles of DEPs under RBSDV infection

**Figure 4:**
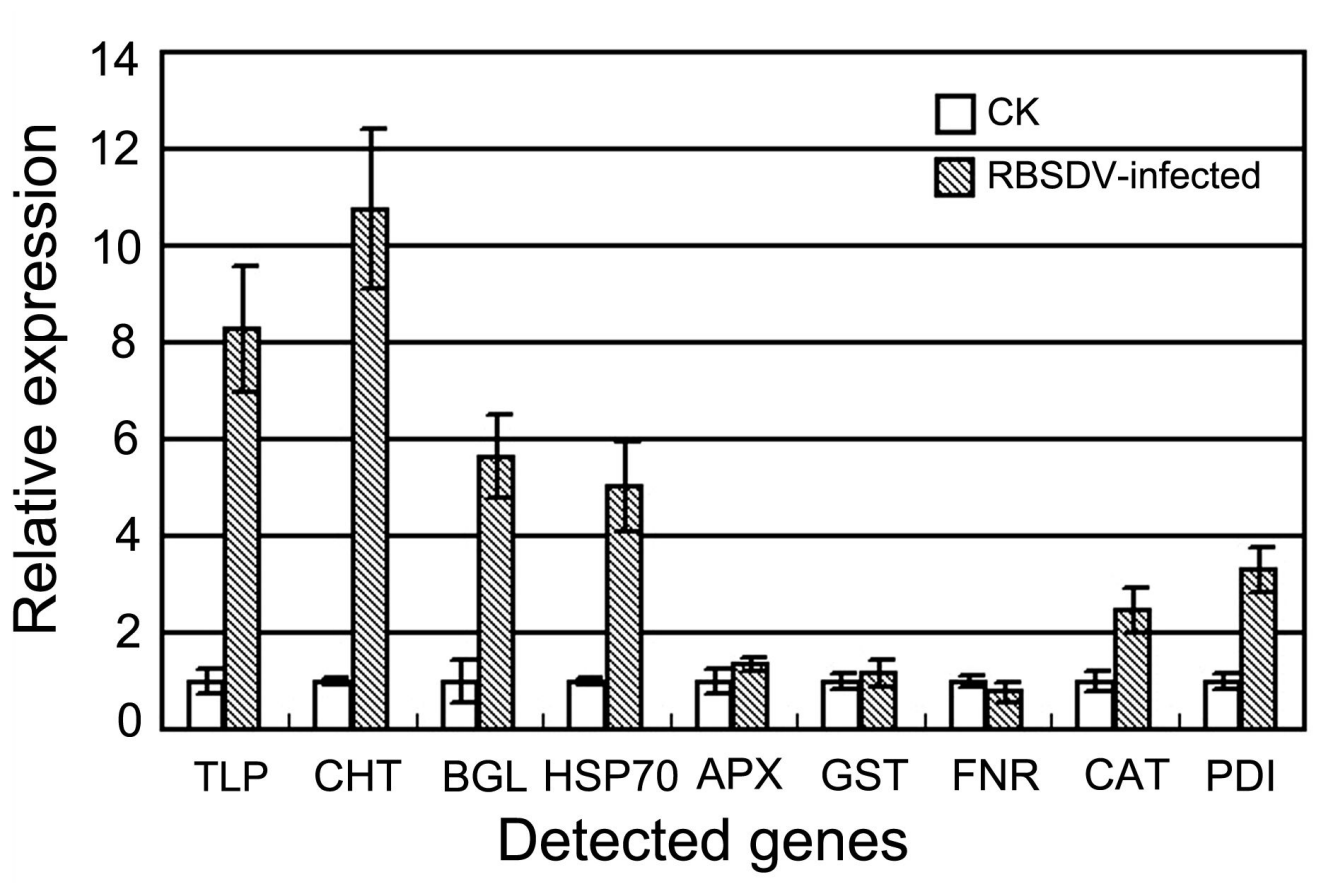
The changes in transcription of the differentially expressed proteins in RBSDV-infected rice. TLP, thaumatin-like protein; CHL, putative chitinase; BGL, beta-1,3-glucanase precursor; HSP70, chloroplast heat shock protein 70; APX1, L-ascorbate peroxodase 1; GST, glutathione S-transferase; FNR, ferredoxin-NADP(H) oxidoreductase; CAT, catalase; PDI, protein disulfide isomerase.

To assess the validity of the alterations in protein expression during RBSDV infection, the transcriptional levels of the mRNA corresponding to the identified proteins were analyzed by quantitative real-time PCR. Nine genes, including four genes related to defense and stress response and five genes related to redox homeostasis, were selected for analysis. Transcription of the genes associated with defense and stress, including thaumatin-like protein (TLP, P01), putative chitinase (CHT, spot 08), beta-1,3-glucanase (BGL, spot 17) and chloroplast heat shock protein 70 (HSP70, spot 39), were elevated after virus infection (Figure 4). The changes in mRNA abundance of these genes were similar to those of their corresponding proteins in the 2-DE gels. The expression patterns of five genes related to redox homeostasis including L-ascorbate peroxodase 1 (APX1, spot 04), glutathione S-transferase (GST, spot 05), ferredoxin-NADP(H) oxidoreductase (FNR, spot 14), catalase (CAT, spot 21) and protein disulfide isomerase (PDI, spot 40) have the same changes as their corresponding proteins (Figure 4). The results suggested that the changes in protein expression detected by proteomic analysis reflect the actual alterations in mock-inoculated and RBSDV-infected plants.

### The photosynthetic response under RBSDV infection stress

**Figure 5:**
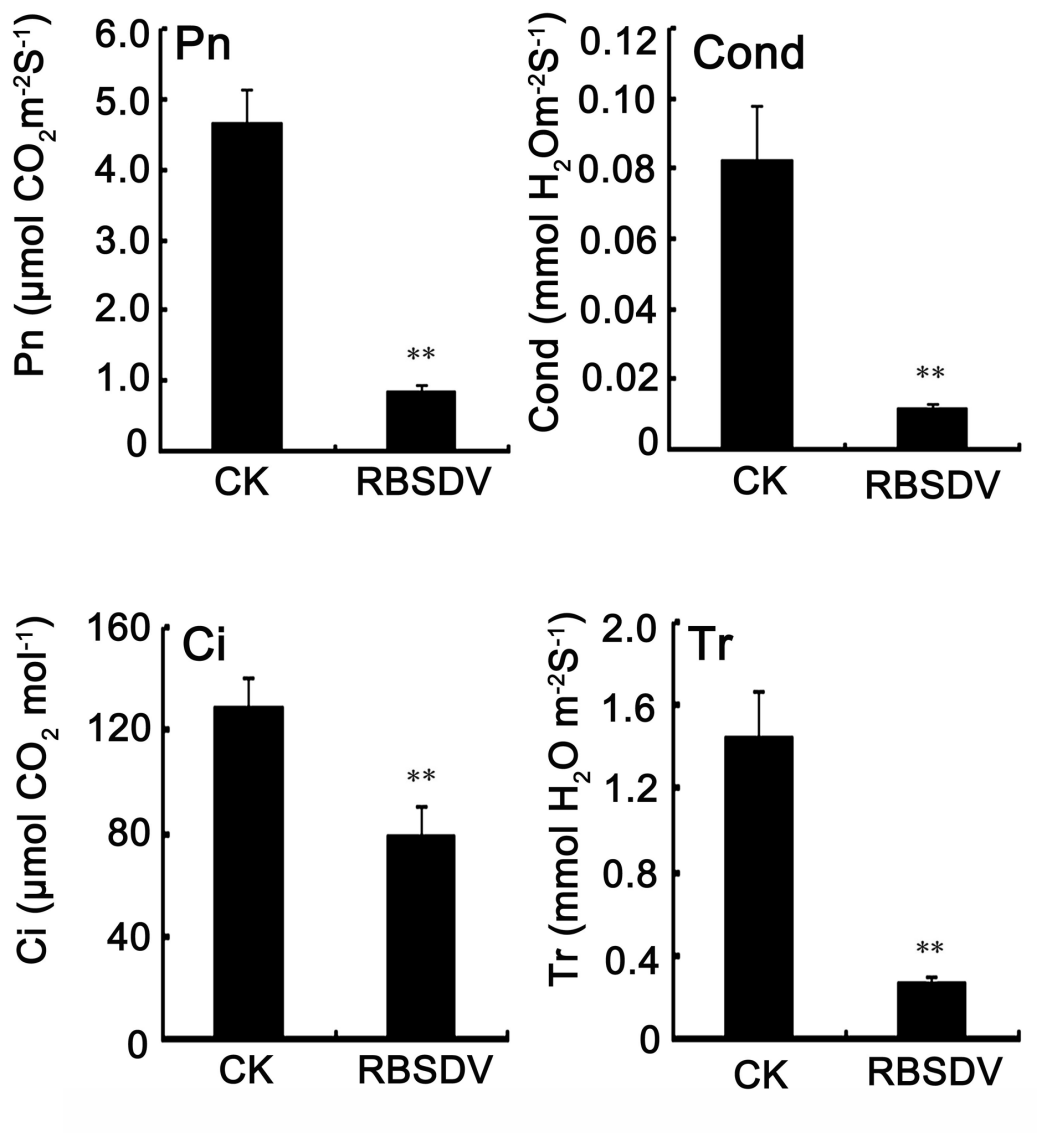
Effects of RBSDV infection on the Pn, Cond, Ci and Tr in rice plant at 50 dpi.

Photosynthesis is a process that converts carbon dioxide into organic compounds and plays a pivotal role in plant growth. In this study, 15 differentially expressed spots (21.7%) were associated with a photosynthetic response, and most of them were down-regulated (Table 1). To investigate the effects of virus infection on rice photosynthesis, the photosynthetic capacity was measured. The net photosynthetic rate (Pn) of the mock-infected rice was 4.68 μmol of CO_2_ m^-2^ s^-1^ at 50 dpi, and the rate for the RBSDV-infected rice was 0.86 μmol of CO_2_ m^-2^ s^-1^ (Figure 5). The stomatal conductance (Cond), the intercellular CO_2_ concentration (Ci) and the transpiration speed (Tr) were also significantly decreased after RBSDV infection (Figure 5). The data above indicated that the RBSDV infection results in a decrease of photosynthesis in rice.

## Discussion

### The overproduced H_2_O_2_ and plant growth deficiency

With both reducing and oxidizing properties, H_2_O_2_ at high concentrations could cause oxidative stress and cell damage. H_2_O_2_ can cross membranes via aquaporin-mediated transport to chloroplasts, mitochondria and cytosol [35] and damage a large variety of biomolecules such as lipids, proteins, and nucleic acids that are essential to the activity and integrity of the cell [36]. Electron microscopy of ultrathin sections data, detected in maize plant infected with *Maize rough dwarf virus* which was later proved to be RBSDV [15,16,37], showed that RBSDV infection destroyed the membrane systems of various cell organelles including chloroplast, mitochondria, nucleus and vacuole, albeit no virus was discovered in these organelles [37]. The accumulated H_2_O_2_ in RBSDV infected plant may harm the membrane structures of various organelles and result in the cell membrane rupture. The H_2_O_2_ content in the virus-susceptible plants was observed increased much higher than in the resistant cultivar [14]. Only in the susceptible plants was the increase in apoplastic H_2_O_2_ levels accompanied by an increase in electrolyte leakage [14]. The H_2_O_2_ at a high level in the virus-susceptible plants probably resulted in the cell damage and electrolyte leakage.

Hydroxyproline-rich glycoprotein-like protein (HRGP) is one of the major classes of structural cell wall proteins and is involved in cell elongation [38,39]. The ROS could limit cell elongation by oxidative crosslink of HRGPs [39]. Previously report showed that cells infected by RBSDV in maize were shorter than those in the mock-inoculated plants [40]. The expression of HRGP was down-regulated by both H_2_O_2_ stress and RBSDV infection (Table 2), suggesting that the H_2_O_2_ overproduced in RBSDV infection may crosslink the HRGP, inhibit the cell elongation, and finally result in the dwarf plant.

### The cross-talk on photosynthesis

The long-term RBSDV accumulation in rice leads to the increase of endogenous H_2_O_2_ and the decrease of photosynthetic parameters, including Pn, Cond, Ci, and Tr (Figure 1 and Figure 5). These phenomena are similar to that obtained in rice under H_2_O_2_ stress. An increase in the chloroplastic hydrogen peroxide levels and an alteration in chloroplast ultrastructure was also observed after Plum pox virus infection [13]. It is presumed that the increased H_2_O_2_ may do damage to the chloroplast ultrastructure and result in the decreased photosynthesis.

Besides, among the seven DEPs related to photosynthesis, six proteins were also responsive to H_2_O_2_ treatment in rice (Table 2). These data indicated that the overproduction H_2_O_2_ had a pivotal role in regulating the process of photosynthesis. The increased H_2_O_2_ under long-term virus infection may affect photosynthesis by regulating proteins in three subgroups, including the light-harvesting reaction, the activation reaction of Rubisco large subunit, and the Calvin cycle.

In the first subgroup, chlorophyll a/b binding protein was down regulated in response to RBSDV infection and H_2_O_2_ stress. Chlorophyll a/b binding protein is a component of the light-harvesting complex of photosystem I and II and facilitates light absorption and energy transfer [41,42]. The decrease in abundance of the protein may inhibit the light absorption and energy transfer and further affect the photosynthetic process in the RBSDV-infected plant. 

In the second subgroup, the Rubisco activase small isoform precursor was up- and down-regulated under both RBSDV infection and H_2_O_2_ stress (Table 2). Rubisco activase was engaged in the activation reaction of Rubisco large subunit. It binds to the inactive Rubisco, facilitates the ATP-dependent removal of sugar phosphates from Rubisco active sites, and maintains Rubisco in its active configuration [43]. The expression change of this protein may severely affect the activation of Rubisco. 

In the third subgroup, four proteins involved in Calvin cycle were co-regulated by RBSDV infection and H_2_O_2_ stress (Table 2). Of them, the abundance of glyceraldehyde-3-phosphate dehydrogenase A, fructose-bisphosphate aldolase and phosphoribulokinase (PRKA) dropped in virus infected plant, implying that the CO_2_ assimilation might be slowed down by RBSDV infection. Glyceraldehyde-3-phosphate dehydrogenase A and fructose-bisphosphate aldolase had the same expression patterns when they were under H_2_O_2_ stress and RBSDV infection. In contrast, the expression patterns of PRKA and Rubisco large subunit were opposite under these two stresses. It suggested that the Calvin cycle was probably disturbed by factors other than H_2_O_2_. PRKA catalyzes the ATP-dependent phosphorylation of ribulose 5-phosphate to form ribulose-1,5-bisphosphate (RuBp) [44]. It was reported that a decrease in Rubisco activity and RuBp regeneration rate were associated with the decreased photosynthesis [45,46]. The decline in Rubisco activity and RuBp regeneration, yet not in the quantity of Rubisco, may result in the decreased in CO_2_ assimilation of rice under RBSDV infection.

Taken together, our results suggested that the overproduction of H_2_O_2_ may diminish light absorption, CO_2_ assimilation and Rubisco activity by modulating the expression of the proteins pertaining to photosynthesis, and finally impaired the photosynthesis. The finding reported above provided new insights into the relationship between the decreased photosynthesis and the overproduction of H_2_O_2_ in RBSDV-infected rice.

### The redox reaction under virus infection and H_2_O_2_ stress

The level of H_2_O_2_ was enhanced after virus accumulation, indicating that an oxidative stress was caused by virus infection. Besides, a series of redox related proteins were differentially expressed under RBSDV infection (Table 1). The significant increase in abundance of APX, CAT and peroxidase (POD) suggested that the H_2_O_2_-scavenging system was activated to reduce the intracellular H_2_O_2_ levels and the oxidative damage. Consistent with this observation, the increase in catalase activity, peroxidase activity and SOD activity was observed after virus inoculation [12,14]. However, the upregulation of these H_2_O_2_-scavenging enzymes seemed to fail in regulating the H_2_O_2_ homeostasis, as an increase of H_2_O_2_ could be observed in virus infected rice (Figure 1).

Under RBSDV infection, 13.0% (nine proteins) of the DEPs were related to redox homeostasis. Three of these proteins were also differentially expressed under H_2_O_2_ stress (Table 2), indicating that the overproduced H_2_O_2_ has a direct effect on plant antioxidative response. APX, a major ROS-scavenging enzyme, could efficiently decompose hydrogen peroxide in various subcellular compartments [47]. GST catalyzes the reduction of toxic organic hydroperoxides with glutathione as a cosubstrate or coenzyme [48]. PDI is participated in the thioredoxin-based redox pathway and the antioxidative defense system [49]. The upregulation of these proteins implied that the host plant provoked the antioxidative response to reduce the toxicity of the accumulated H_2_O_2_ after virus infection.

### The effect of overproduced H_2_O_2_ on the other processes

Proteins involved in carbohydrate metabolism, amino acid metabolism, nucleotide metabolism, energy pathway, and transcription and translation were regulated by both H_2_O_2_ and RBSDV stresses (Table 2), suggesting that the enhanced H_2_O_2_ in RBSDV-infected plant may disturb these processes.

Fructokinase catalyzes the transfer of a phosphate group from ATP to fructose and plays a role in the glycolysis, in particular, sucrose and fructose metabolism. *Fructokinase 2* could inhibit the growth of stems and roots in tomato when it was suppressed [50]. The abundance of fructokinase 2 was decreased under H_2_O_2_ stress, but was up- and down-regulated under RBSDV infection (Table 2). The multiple expression patterns indicated that fructokinase 2 may impair the glycolysis and inhibit the plant growth in disease plant.

ADP-glucose pyrophosphorylase (AGPase) activates the first, rate limiting step in starch biosynthesis. UDP-glucose pyrophosphorylase could catalyze UDP-glucose into glucose-1-phosphate (Glc-1-P),which can be converted into ADP-glucose by AGPase and finally stored as starch [51]. The upregulation of AGPase and UDP-glucose pyrophosphorylase suggested that more starch might be accumulated under these stresses, which may explain why starch granules boomed in the maize leaves infected with RBSDV [37]. 

The RNA binding protein play a major role in post-transcriptional regulation, including pre-mRNA splicing, capping, mRNA transport and translation of functional mRNAs [52]. Under RBSDV infection and H_2_O_2_ stress, some responsive proteins had different isoelectric points and different expression patterns (Table 1). We reasoned that mRNA binding protein is likely involved in posttranslational regulation and results in the opposite expression patterns of the responsive proteins.

## Conclusions

In the current study, a number of proteins responsive to both H_2_O_2_ stress and long-term RBSDV infection were obtained. These proteins were associated with various functions, including photosynthesis, redox homeostasis, energy pathway, carbohydrate, amino acid, and nucleotide metabolism, energy pathway, transcription and translation, and cell wall modification. The results should be useful in providing insights into the significant role of H_2_O_2_ produced in plant-virus compatible interaction. It is worth noting that no PR proteins could be up-regulated by H_2_O_2_ stress. The function of H_2_O_2_ overproduced in compatible interaction seems different from that revealed in incompatible interaction. Whether the overproduced H_2_O_2_ facilitates the RBSDV infection remains to be further studied.

## Supporting Information

Table S1 **Primers used for RT-PCR and quantitative real-time PCR.**
(DOC)

Table S2 **The proteins were annotated by BLASTP (www.ncbi.nlm.nih.gov/BLAST and www.uniprot.org/BLAST).** The homologues with the highest homology are shown.
(DOC)

Table S3 **Peptide sequences identified by MOLDI-TOF/TOF-MS.**
(DOC)
