# Supplementary material for: Comparative Proteomic Analysis Reveals the Cross-Talk between the Responses Induced by H2O2 and by Long-Term Rice Black-Streaked Dwarf Virus Infection in Rice
Source: PLoS One. 2013 Nov 27;8(11):e81640. doi: 10.1371/journal.pone.0081640 (PMC3842349; doi:10.1371/journal.pone.0081640)
Supplement: Table S1 — Primers used for RT-PCR and quantitative real-time PCR. (DOC) [file pone.0081640.s001.doc]

Table S1. Primers used for RT-PCR and quantitative real-time PCR

| **Spots no** | **Gene name** | **Accession number** | **Primer sequences (5’→3’)** |
| --- | --- | --- | --- |
|  | RBSDV S9-1 | NC_003731 | P9-1-F: ATGGCAGACCAAGAGCGGAG |
|  |  |  | P9-1-R: TCAAACGTCCAATTTCAAGG |
|  | RBSDV S10 | NC_003733 | P10-F: ATGGCTGACATAAGACTCGA |
|  |  |  | P10-R: TCATCTTGTCACTTTGTTTA |
| 01 | Thaumatin-like pathogenesis-related protein 3 precursor | X68197 | F: GACGCTGGCGGAGTTCAC |
|  |  |  | R: AAGCTCATGGCGACGTTGTAG |
| 08 | Putative chitinase | NM_001061552 | F: TACGTGTCGCACAAGGATCTCT |
|  |  |  | R: TCTTGTCCCAGTACCTGTTCCA |
| 17 | Beta-1,3-glucanase precursor | NM_001051892 | F: CAGCGGTTCAGTCCATTGG |
|  |  |  | R: ATGTCGCTCCTCGGGAAGTA |
| 9 | Chloroplast heat shock protein 70 | NM_001061675 | F: AGGCGATGTGAAGGGTAAGGT |
|  |  |  | R: GCACCCTGCTGGCTGTAGAG |
| 04 | Ascorbate peroxidase | AY254495 | F: ACCCAGGAAGGGAGGACAAA |
|  |  |  | R: AGGGCAACAATGTCCTGATCA |
| 05 | Putative glutathione S-transferase | AF402804 | F: AGCACGGCTACCCCATCA |
|  |  |  | R: CGTCATGAAGATCGGGTACAGA |
| 14 | Ferredoxin-NADP(H) oxidoreductase | AK065309 | F: CCAACGCCACCATCATCAT |
|  |  |  | R: TCGTCGTGCTCCTCGAAGA |
| 21 | Catalase | EF371902 | F: CAACCGCAACGTCGACAA |
|  |  |  | R: TCGGCGTACGCGAACAC |
| 40 | Protein disulfide isomerase | AY987391 | F: CGTCACCCCCAGTGGAAAG |
|  |  |  | R: GCCGACTCTGCCTTCTCCTT |
|  | OsUBQ5 | AK061988 | F: ACCACTTCGACCGCCACTACT |
|  |  |  | R: ACGCCTAAGCCTGCTGGTT |
