# Supplementary material for: Comparative Proteomic Analysis Reveals the Cross-Talk between the Responses Induced by H2O2 and by Long-Term Rice Black-Streaked Dwarf Virus Infection in Rice
Source: PLoS One. 2013 Nov 27;8(11):e81640. doi: 10.1371/journal.pone.0081640 (PMC3842349; doi:10.1371/journal.pone.0081640)
Supplement: Table S2 — The proteins were annotated by BLASTP (www.ncbi.nlm.nih.gov/BLAST and www.uniprot.org/BLAST). The homologues with the highest homology are shown. (DOC) [file pone.0081640.s002.doc]

Table S2. The proteins were annotated by BLASTP ([www.ncbi.nlm.nih.gov/BLAST](http://www.ncbi.nlm.nih.gov/BLAST) and [www.uniprot.org](http://www.uniprot.org/)/BLAST). The homologues with the highest homology are shown.

| **Spot no.** | **Protein name** | **Homologue** | | | | | |
| --- | --- | --- | --- | --- | --- | --- | --- |
|  |  | **Accession no.** | **Protein name** | **Protein accession no.** | **Identities (%)** | **E- value** | **Organism** |
| 01 | Os12g0628600 | gi|115489688 | Thaumatin-like protein | [P31110](http://www.uniprot.org/uniprot/P31110) | 100 | 1e-100 | *O.Sativa* |
| 02 | hypothetical protein OsI_22755 | gi|125555124 | Chlorophyll a/b binding protein | [Q9ZSU0](http://www.uniprot.org/uniprot/Q9ZSU0) | 99 | 1e-140 | *O.Sativa* |
| 05 | Os09g0467200 | gi|115479659 | Glutathione transferase GST 23 | BAD17523 | 100 | 8e-162 | *O.Sativa* |
| 07 | OsI_08842 | gi|218191527 | Glutamine synthetase | [B8AI28](http://www.uniprot.org/uniprot/B8AI28) | 100 | 0 | *O.Sativa* |
| 13 | OSIGBa0076I14.3 | gi|116309406 | Abscisic stress ripening protein | [Q41300](http://www.uniprot.org/uniprot/Q41300) | 44 | 4e-42 | *O.Sativa* |
| 15 | Os02g0240300 | gi|115445243 | Class III peroxidase 29 | [Q6ER49](http://www.uniprot.org/uniprot/Q6ER49) | 100 | 0 | *O.Sativa* |
| 18 | Os08g0113100 | gi|115474481 | Fructokinase-2 | [Q0J8G4](http://www.uniprot.org/uniprot/Q0J8G4) | 100 | 0 | *O.Sativa* |
| 22 | Os07g0513000 | gi|115472339 | ATP synthase gamma chain | [Q84NW1](http://www.uniprot.org/uniprot/Q84NW1) | 100 | 0 | *O.Sativa* |
| 26 | Os02g0698000 | gi|115448091 | Phosphoribulokinase | [Q8GRU9](http://www.uniprot.org/uniprot/Q8GRU9) | 100 | 0 | *O.Sativa* |
| 27 | Os02g0519900 | gi|115446385 | Elongation factor 2 | [Q6H4L2](http://www.uniprot.org/uniprot/Q6H4L2) | 100 | 0 | *O.Sativa* |
| 33 | OsI_20474 | gi|125552851 | Phosphoglycerate kinase | [A2Y650](http://www.uniprot.org/uniprot/A2Y650) | 100 | 1e-153 | *O.Sativa* |
| 38 | Os01g0947000 | gi|115442217 | Endo-1,3-beta-glucanase | BAC15778 | 100 | 0 | *O.Sativa* |
| 39 | Os05g0303000 | gi|115463081 | Chloroplast heat shock protein 70 | [ABP65327](http://www.ncbi.nlm.nih.gov/protein/145388994?report=genbank&log$=protalign&blast_rank=2&RID=PPA50X2B013) | 94 | 0 | *Cenchrus americanus* |
| 41 | Os02g0257300 | gi|115445387 | Hydroxyproline-rich glycoprotein-like | [Q6ETQ7](http://www.uniprot.org/uniprot/Q6ETQ7) | 100 | 0 | *O.Sativa* |
| 42 | Os06g0725900 | gi|115470052 | ATP-dependent zinc metalloprotease FTSH 1 | [Q5Z974](http://www.uniprot.org/uniprot/Q5Z974) | 100 | 0 | *O.Sativa* |
| 43 | Os04g0659300 | gi|115461070 | Receptor-like protein kinase DUF26 | [AAQ19329](http://www.uniprot.org/uniprot/Q8S3P3) | 100 | 0 | *O.Sativa* |
| 44 | hypothetical protein OsI_33583 | gi|125531926 | Putative class III chitinase | [Q8S870](http://www.uniprot.org/uniprot/Q8S870) | 99 | 1e-165 | *O.Sativa* |
| 45 | Os08g0113100 | gi|115474481 | Fructokinase-2 | [Q0J8G4](http://www.uniprot.org/uniprot/Q0J8G4) | 100 | 0 | *O.Sativa* |
| 47 | hypothetical protein | gi|19386746 | Unknown protein | BAB86127 | 100 | 0 | *O.Sativa* |
| 55 | Os08g0536000 | gi|115477529 | Pyruvate dehydrogenase E1 component subunit beta | Q6Z1G7 | 100 | 0 | *O.Sativa* |
| 59 | Os07g0212200 | gi|115471157 | putative mRNA binding protein precursor | BAC83225 | 100 | 0 | *O.Sativa* |
| 61 | Os02g0192700 | gi|115444771 | Thioredoxin peroxidase | BAD15391 | 100 | 2e-156 | *O.Sativa* |
| 62 | Os01g0233000 | gi|115435500 | Salt stress root protein RS1 | Q0JPA6 | 100 | 1e-107 | *O.Sativa* |
| 65 | Os04g0459500 | gi|115458768 | Glyceraldehyde-3-phosphate dehydrogenase A | [P09315](http://www.uniprot.org/uniprot/P09315) | 95 | 0 | Zea mays |
| 66 | OsI_20474 | gi|125552851 | Phosphoglycerate kinase | [A2Y650](http://www.uniprot.org/uniprot/A2Y650) | 94 | 1e-153 | *O.Sativa* |
| 69 | Os01g0233000 | gi|115435500 | Salt stress root protein RS1 | [Q0JPA6](http://www.uniprot.org/uniprot/Q0JPA6) | 100 | 1e-107 | *O.Sativa* |
| 70 | Os04g0490800 | gi|115459134 | 4-nitrophenylphosphatase | [B6UCU8](http://www.uniprot.org/uniprot/B6UCU8) | 86 | 1e-174 | O.Sativa |
| 72 | Os07g0513000 | gi|115472339 | ATP synthase gamma chain | [Q84NW1](http://www.uniprot.org/uniprot/Q84NW1) | 100 | 0 | O.Sativa |
