# Supplementary material for: Comparative Proteomic Analysis Reveals the Cross-Talk between the Responses Induced by H2O2 and by Long-Term Rice Black-Streaked Dwarf Virus Infection in Rice
Source: PLoS One. 2013 Nov 27;8(11):e81640. doi: 10.1371/journal.pone.0081640 (PMC3842349; doi:10.1371/journal.pone.0081640)
Supplement: Table S3 — Peptide sequences identified by MOLDI-TOF/TOF-MS. (DOC) [file pone.0081640.s003.doc]

Table S3. Peptide sequences identified by MOLDI-TOF/TOF-MS.

| **Spot no.** | **Protein name** | **Accession no** | **Peptide sequence** |
| --- | --- | --- | --- |
| 01 | Thaumatin-like protein | gi|115489688 | CPDAYLFPEDNTK |
|  |  |  | CSFTVWPAATPVGGGVQLSPGQTWTINVPAGTSSGR |
| 02 | Chlorophyll a/b binding protein | gi|125555124 | KYPGGAFDPLGFSK |
| 04 | L-ascorbate peroxodase 1, cytosolic | gi|158512874 | TPAELSHAANAGLDIAVR |
|  |  |  | QVFGAQMGLSDQDIVALSGGHTLGR |
| 05 | Glutathione transferase GST 23 | gi|115479659 | KLVHEAQQCLK |
|  |  |  | HGYPIMPSDPFDR |
|  |  |  | GVDYEYVDEDLANK |
|  |  |  | CNAALYPIFMTTGEEQR |
|  |  |  | KVPVLVHDGKPLAESTVIVEYIDEAWK |
| 06 | Chlorophyll a/b binding protein | gi|108864186 | YQAYELIHAR |
|  |  |  | FGANCGPEAVWFK |
|  |  |  | WAMLGAAGFIIPEACNK |
|  |  |  | HLSDPFGNNLLTVISGAAER |
|  |  |  | LHPGGPFDPLGLASDPDQAALLK |
|  |  |  | SEVPDYLNGEVPGDYGYDPFGLSK |
| 07 | Glutamine synthetase | gi|218191527 | EHISAYGEGNER |
|  |  |  | HKEHISAYGEGNER |
|  |  |  | HETADINTFSWGVANR |
| 08 | Putative chitinase | gi|54291729 | LQAALSTGLFSR |
|  |  |  | NTDQLEGSLR |
|  |  |  | LQAALSTGLFSR |
|  |  |  | VLVGVVASPEADR |
|  |  |  | LPNYGGIMVWNR |
|  |  |  | DLYYDVLQFINK |
|  |  |  | LDISGHTVSAVGPDIK |
|  |  |  | VLVGVVASPEADRDGYVSHK |
| 09 | Putative chitinase | gi|54291729 | LQAALSTGLFSR |
|  |  |  | VLVGVVASPEADR |
|  |  |  | LPNYGGIMVWNR |
|  |  |  | DLYYDVLQFINK |
|  |  |  | VLVGVVASPEADRDGYVSHK |
| 10 | Putative chitinase | gi|54291729 | LQAALSTGLFSR |
|  |  |  | VLVGVVASPEADR |
| 11 | Putative chitinase | gi|54291729 | LQAALSTGLFSR |
|  |  |  | VLVGVVASPEADR |
|  |  |  | LPNYGGIMVWNR |
|  |  |  | DLYYDVLQFINK |
|  |  |  | LDISGHTVSAVGPDIK |
|  |  |  | VLVGVVASPEADRDGYVSHK |
| 12 | Putative chitinase | gi|54291729 | LQAALSTGLFSR |
|  |  |  | VLVGVVASPEADR |
|  |  |  | LPNYGGIMVWNR |
|  |  |  | DLYYDVLQFINK |
|  |  |  | LDISGHTVSAVGPDIK |
|  |  |  | VLVGVVASPEADRDGYVSHK |
| 13 | Abscisic stress ripening protein | gi|116309406 | SGTDDDYDSGYNNR |
|  |  |  | LGEVGALAAGAFAMYER |
|  |  |  | RPGGGAGGYNKPGGTDDYDSGYNK |
|  |  |  | SGDDEYSGGGGAEADEEYVDGLSSR |
| 14 | Putative ferredoxin-NADP(H) oxidoreductase | gi|41052915 | DGIDWLDYKK |
|  |  |  | GIDDIMIDLAAK |
|  |  |  | KSEQWNVEVY |
|  |  |  | LVYTNDQGEIVK |
|  |  |  | EGQSIGVIPDGIDK |
|  |  |  | RLVYTNDQGEIVK |
|  |  |  | LYSIASSAIGDFADSK |
|  |  |  | MAEYKDELWELLK |
|  |  |  | GVCSNFLCDLKPGSDVK |
|  |  |  | DPNATIIMLGTGTGIAPFR |
|  |  |  | ITGDDAPGETWHMVFSTDGEIPYR |
| 15 | Class III peroxidase 29 precursor | gi|115445243 | MGNIGQPSDGEVR |
|  |  |  | DSTALLGGPSWAVPLGR |
|  |  |  | AHIYNDANIDPSFAALR |
|  |  |  | GLLHSDQVLFNGGSQDALVR |
|  |  |  | DMTALSGSHTVGFSQCTNFR |
| 16 | Translational elongation factor Tu | gi|17225494 | KYDEIDAAPEER |
|  |  |  | GITINTATVEYETETR |
|  |  |  | DQVDDEELLQLVELEVR |
|  |  |  | KDQVDDEELLQLVELEVR |
| 17 | Beta-1,3-glucanase precursor | gi|4097942 | QNVQAYPGVSFR |
|  |  |  | YIAVGNEVTGDDTGNILPAMK |
| 18 | Fructokinase-2 | gi|115474481 | LPLWPSEDAAR |
|  |  |  | NVLSLWFDGLK |
|  |  |  | FSNACGAICTTK |
|  |  |  | SAGILCSYDPNVR |
|  |  |  | FGDDEFGHMLVDILK |
|  |  |  | IFHYGSISLITEPCR |
|  |  |  | VSDDEVAFLTQGDANDEK |
|  |  |  | NPSADMLLTEAELNLDLIR |
| 19 | Fructose-bisphosphate aldolase | gi|108864048 | ATPEQVSDYTLK |
|  |  |  | LASIGLENTEANR |
|  |  |  | TVVSIPNGPSELAVK |
|  |  |  | GLVPLAGSNNESWCQGLDGLASR |
|  |  |  | YAAISQDNGLVPIVEPEILLDGEHGIDR |
| 20 | 5-methyltetrahydropteroyltriglutamate-homocysteine methyltransferase | gi|108862990 | FETCYQIALAIK |
|  |  |  | YGAGIGPGVYDIHSPR |
|  |  |  | KAEHAFYLDWAVHSFR |
|  |  |  | IQEELDIDVLVHGEPER |
|  |  |  | EVEDLEAGGIQVIQIDEAALR |
| 21 | Catalase | gi|283050393 | FPDVIHAFKPNPR |
|  |  |  | TWPEDEVPLRPVGR |
|  |  |  | HMDGFGVNTYTFVTR |
|  |  |  | DGIKFPDVIHAFKPNPR |
|  |  |  | EGNWDLLGNNFPVFFIR |
|  |  |  | TTTTNAGAPVWNDNEALTVGPR |
|  |  |  | GFFECTHDVTDITCADFLR |
|  |  |  | NHSHATQDLYDSIAAGNFPEWK |
|  |  |  | LFVQVIDPEEEERFDFDPLDDTK |
| 22 | ATP synthase gamma chain | gi|115472339 | GLCGSFNNNVLK |
|  |  |  | ALQESLASELAAR |
|  |  |  | QLGLEYTVVSVGK |
|  |  |  | SDPIIQTLLPMSPK |
| 23 | ATP synthase beta subunit | gi|56784991 | AHGGFSVFAGVGER |
|  |  |  | VGLTGLTVAEHFR |
|  |  |  | FTQANSEVSALLGR |
|  |  |  | CALVYGQMNEPPGAR |
|  |  |  | DAEGQDVLLFIDNIFR |
|  |  |  | QISELGIYPAVDPLDSTSR |
|  |  |  | IPSAVGYQPTLATDLGGLQER |
| 24 | ATP synthase CF1 beta subunit | gi|50233978 | AHGGVSVFGGVGER |
|  |  |  | IVGNEHYETAQR |
|  |  |  | FVQAGSEVSALLGR |
|  |  |  | VGLTALTMAEYFR |
|  |  |  | VALVYGQMNEPPGAR |
|  |  |  | QINVTCEVQQLLGNNR |
|  |  |  | DVNKQDVLLFIDNIFR |
|  |  |  | GIYPAVDPLDSTSTMLQPR |
|  |  |  | GMEVIDTGAPLSVPVGGATLGR |
|  |  |  | MPSAVGYQPTLSTEMGSLQER |
| 25 | ATP synthase CF1 beta subunit | gi|50233978 | AHGGVSVFGGVGER |
|  |  |  | IVGNEHYETAQR |
|  |  |  | FVQAGSEVSALLGR |
|  |  |  | VGLTALTMAEYFR |
| 26 | Phosphoribulokinase | gi|115448091 | FYGEVTQQMLK |
|  |  |  | KPDFDAFIDPQK |
|  |  |  | IRDLYEQIIAER |
|  |  |  | IFVIEGLHPMFDER |
|  |  |  | DLLDFSIYLDISDEVK |
|  |  |  | VRDLLDFSIYLDISDEVK |
|  |  |  | QYADAVIEVLPTQLIPDDNEGK |
|  |  |  | NFNPVYLFDEGSSITWVPCGR |
|  |  |  | AIEKPIYNHVTGLLDPPELIQPPK |
|  |  |  | FAYGPDTYFGHEVSVLEMDGQFDR |
|  |  |  | GGNPDSNTLISDTTTVICLDDYHSLDR |
| 27 | Elongation factor 2 | gi|115446385 | GFVQFCYEPIK |
|  |  |  | LWGENFFDPATK |
|  |  |  | STGISLFYEMSDESLK |
|  |  |  | VENLYEGPLDDVYATAIR |
|  |  |  | CFLELQVEGEEAYQTFSR |
|  |  |  | STLTDSLVAAAGIIAQEVAGDVR |
|  |  |  | YRVENLYEGPLDDVYATAIR |
|  |  |  | ITDGALVVVDCIEGVCVQTETVLR |
|  |  |  | DGNEYLINLIDSPGHVDFSSEVTAALR |
| 28 | RuBisCO activase small isoform precursor | gi|8918361 | VYDDEVRK |
|  |  |  | SFQCELVFAK |
|  |  |  | FYWAPTRDDR |
|  |  |  | WVSDTGVENIGKR |
|  |  |  | GLAYDISDDQQDITR |
|  |  |  | IVDSFPGQSIDFFGALR |
|  |  |  | VPIIVTGNDFSTLYAPLIR |
| 29 | RuBisCO activase small isoform precursor | gi|8918361 | SFQCELVFAK |
|  |  |  | FYWAPTRDDR |
|  |  |  | WVSDTGVENIGKR |
|  |  |  | GLAYDISDDQQDITR |
|  |  |  | MCCLFINDLDAGAGR |
|  |  |  | IVDSFPGQSIDFFGALR |
| 30 | RuBisCO activase small isoform precursor | gi|62733297 | GLAYDISDDQQDITR |
|  |  |  | MCCLFINDLDAGAGR |
|  |  |  | IVDSFPGQSIDFFGALR |
|  |  |  | VPIIVTGNDFSTLYAPLIR |
| 31 | RuBisCO activase small isoform precursor | gi|8918361 | FYWAPTR |
|  |  |  | VYDDEVRK |
|  |  |  | SFQCELVFAK |
|  |  |  | WVSDTGVENIGKR |
|  |  |  | GLAYDISDDQQDITR |
|  |  |  | IVDSFPGQSIDFFGALR |
|  |  |  | VPIIVTGNDFSTLYAPLIR |
| 32 | Enolase | gi|110288667 | IEEELGAAAVYAGAK |
|  |  |  | VNQIGSVTESIEAVK |
|  |  |  | TYDLNFKEENNDGSQK |
|  |  |  | SGETEDTFIADLAVGLATGQIK |
|  |  |  | YGQDATNVGDEGGFAPNIQENK |
|  |  |  | MTAEIGEQVQIVGDDLLVTNPTR |
|  |  |  | SFVSEYPIVSIEDPFDQDDWEHYAK |
| 33 | Putative chloroplast phosphoglycerate kinase | gi|46981258 | ELDYLVGAVSSPK |
|  |  |  | FLKPSVAGFLLQK |
|  |  |  | LASLADLYVNDAFGTAHR |
|  |  |  | ADLNVPLDDNQNITDDTR |
|  |  |  | KLASLADLYVNDAFGTAHR |
| 34 | chloroplast translational elongation factor Tu | gi|6525065 | KYDEIDAAPEER |
|  |  |  | TMDDAMAGDNVGLLLR |
|  |  |  | GITINTATVEYETETR |
|  |  |  | MVVELIQPVACEQGMR |
|  |  |  | DQVDDEELLQLVELEVR |
|  |  |  | QTDLPFLLAVEDVFSITGR |
|  |  |  | KDQVDDEELLQLVELEVR |
|  |  |  | ELLSSYEYDGDEVPIVAGSALK |
|  |  |  | RGDDEWVDGIFSLIDSVDNYIPVPQR |
| 36 | ADP-glucose pyrophosphorylase small subunit | gi|217075932 | AKPAVPLGANYR |
|  |  |  | IINVDNVQEAAR |
|  |  |  | KPVPDFSFYDR |
|  |  |  | AMMVDTTILGLDDVR |
|  |  |  | VLDADVTDSVIGEGCVIK |
|  |  |  | EQFPGANDFGSEVIPGATNIGMR |
|  |  |  | SSQTCLDPDASTSVLGIILGGGAGTR |
|  |  |  | NEGFVEVLAAQQSPDNPNWFQGTADAVR |
| 37 | Ribulose-1,5-bisphosphate carboxylase/oxygenase large subunit | gi|11466795 | EMTLGFVDLLR |
|  |  |  | LTYYTPEYETK |
|  |  |  | TFQGPPHGIQVER |
|  |  |  | GGLDFTKDDENVNSQPFMR |
|  |  |  | VTPQPGVPPEEAGAAVAAESSTGTWTTVWTDGLTSLDR |
| 38 | Endo-1,3-beta-glucanase | gi|115442217 | AYNQGLIDHVGR |
|  |  |  | DNVEAYWPSVIIR |
|  |  |  | DISLNYATFQPGTTVR |
|  |  |  | EISLNYATFQPGTTVR |
|  |  |  |  |
| 39 | Chloroplast heat shock protein 70 | gi|115463081 | DIDEVILVGGSTR |
|  |  |  | AKFEELCSDLLDR |
|  |  |  | SEVFSTAADGQTSVEINVLQGER |
| 40 | Protein disulfide isomerase | gi|62546209 | SDYDFGHTLHANHLPR |
| 41 | Hydroxyproline-rich glycoprotein-like | gi|115445387 | FKDPENTTLVILDK |
|  |  |  | ALVEEPGAQLVDIRPPGDAR |
|  |  |  |  |
| 42 | ATP-dependent zinc metalloprotease FTSH 1 | gi|115470052 | DGGLLQLTAIDGR |
|  |  |  | SYLENQMAVALGGR |
|  |  |  | GQAGGLTFFAPSEER |
|  |  |  | DYSMATADVVDAEVR |
|  |  |  | APCIVFIDEIDAVGR |
|  |  |  | AKAPCIVFIDEIDAVGR |
|  |  |  | VAEEVIFGQENVTTGASNDFMQVSR |
| 43 | Receptor-like protein kinase DUF26 | gi|115461070 | GCLEGLVAR |
|  |  |  | AACYDCGTSVWR |
|  |  |  | DVALVYNECYAR |
|  |  |  | LLAATAEYAAGDIAR |
|  |  |  | WWDTFPANVDGAR |
|  |  |  | AKDVALVYNECYAR |
|  |  |  | SEVYPFYTGAPMVVLR |
| 44 | Putative class III chitinase | gi|125531926 | VSSYGFEYEIK |
|  |  |  | LPGLFIWSADSSK |
|  |  |  | TGDVAGLLSPEQGIAGAK |
|  |  |  | HPNITTSIAPFEDAVVQR |
|  |  |  | AAHPNVSVMVGLGGDSVQDTAK |
| 45 | Fructokinase-2 | gi|115474481 | LPLWPSEDAAR |
|  |  |  | NVLSLWFDGLK |
|  |  |  | SAGILCSYDPNVR |
|  |  |  | FGDDEFGHMLVDILK |
|  |  |  | IFHYGSISLITEPCR |
|  |  |  | VSDDEVAFLTQGDANDEK |
|  |  |  | NPSADMLLTEAELNLDLIR |
| 46 | 5-methyltetrahydropteroyltriglutamate-homocysteine methyltransferase | gi|108862994 | YLFAGVVDGR |
|  |  |  | SSAEDLEKVATDLR |
|  |  |  | TLTSLNSVTAYGFDLIR |
|  |  |  | ALAGQKDEAYFAANAAAQASR |
| 47 | Unkown protein | gi|19386746 | SSWNSPYYDTSSYGAGSGGGGGGGR |
| 48 | r40c1 protein | gi|24899397 | ILPWGDEAYAGGSANAPR |
|  |  |  | HSNSIKDEEGYPAFALVNR |
| 49 | Fructose-bisphosphate aldolase | gi|115463789 | VSPEVIAEYTVR |
|  |  |  | ETTTQGHDDLGKR |
|  |  |  | KVSPEVIAEYTVR |
|  |  |  | FASINVENVEENR |
|  |  |  | CAYVSEVVLAACYK |
|  |  |  | IGPNEPSQLAIDLNAQGLAR |
|  |  |  | ALNEHHVLLEGTLLKPNMVTPGSDAK |
|  |  |  | YAIICQENGLVPIVEPEILVDGPHDIDR |
| 50 | Wheat adenosylhomocysteinase-like protein | gi|29367605 | GETLEEYWWCTER |
|  |  |  | WVFPETNTGIIVLAEGR |
| 51 | Heat shock cognate 70 kDa protein | gi|108864707 | MVNHFVQEFK |
|  |  |  | SSVHDVVLVGGSTR |
|  |  |  | TTPSYVGFTDSER |
|  |  |  | NAVVTVPAYFNDSQR |
|  |  |  | GEGPAIGIDLGTTYSCVGVWQHDR |
|  |  |  | EQVFSTYSDNQPGVLIQVYEGER |
|  |  |  | TLSSTAQTTIEIDSLYEGIDFYSTITR |
| 53 | Ribonuclease 3 precursor | gi|149392262 | YGYPSEDFFVK |
|  |  |  | SEWNSYGVCSGLK |
|  |  |  | SFMTFDSSENTAVVR |
|  |  |  | QLYEIYLCVDKDAK |
|  |  |  | LDSIENNLNHYWSNIK |
|  |  |  | NADVLSALAEQGIKPDYQLYNTAFIK |
| 54 | Ribonuclease 3 precursor | gi|149392262 | YGYPSEDFFVK |
|  |  |  | SEWNSYGVCSGLK |
|  |  |  | SFMTFDSSENTAVVR |
|  |  |  | LDSIENNLNHYWSNIK |
|  |  |  | NADVLSALAEQGIKPDYQLYNTAFIK |
| 55 | Pyruvate dehydrogenase E1 component subunit beta | gi|115477529 | EGISAEVINLR |
|  |  |  | IAGADVPMPYAANLER |
| 56 | P9-1 protein [Rice black streaked dwarf virus] | gi|15387604 | TCYCGFNYSHLPNLER |
|  |  |  | ENFELCTENLDLKDYDR |
|  |  |  | ESEESLSSEILEGEAAVVNVFK |
|  |  |  | EVNHIFYQLATFDNYPFDLLR |
| 57 | ATP synthase CF1 beta subunit | gi|50233978 | AHGGVSVFGGVGER |
|  |  |  | IVGNEHYETAQR |
|  |  |  | FVQAGSEVSALLGR |
|  |  |  | VGLTALTMAEYFR |
|  |  |  | VALVYGQMNEPPGAR |
|  |  |  | QINVTCEVQQLLGNNR |
|  |  |  | DVNKQDVLLFIDNIFR |
|  |  |  | GIYPAVDPLDSTSTMLQPR |
|  |  |  | GMEVIDTGAPLSVPVGGATLGR |
|  |  |  | MPSAVGYQPTLSTEMGSLQER |
|  |  |  | IFNVLGEPVDNLGPVDTSATFPIHR |
| 58 | Carboxyl-terminal peptidase-like | gi|55296403 | DLNTIETGWQVYPAMYGDDK |
|  |  |  | DLNTIETGWQVYPAMYGDDKTR |
|  |  |  | VTINVWQPTIATSGDFSLSQLWISAGSYDNK |
| 59 | Putative mRNA binding protein precursor | gi|115471157 | DCEEWFFDR |
|  |  |  | SSTNLPEDLKER |
|  |  |  | DLDAVKPVVDWAK |
|  |  |  | DLASMVALAVESPGAAAGR |
|  |  |  | DLLAAGHAVTVLTVGDE |
|  |  |  | GRPVPIPGSGMQVTNISHVR |
|  |  |  | MCAAAAGAQPEILHYDPAAVGVDAK |
|  |  |  | AAGVAQFLFVSSAGIYTPSDEPPHVEGDAVK |
| 60 | Fructose-bisphosphate aldolase | gi|108864048 | ATPEQVSDYTLK |
|  |  |  | TVVSIPNGPSELAVK |
|  |  |  | YAAISQDNGLVPIVEPEILLDGEHGIDR |
| 61 | Thioredoxin peroxidase | gi|115444771 | TVTVAELTAGR |
|  |  |  | YALLADDGVVK |
|  |  |  | AVLFAVPGAFTPTCSQK |
|  |  |  | GVDAIACVSVNDAFVMR |
|  |  |  | LPDATLSYFDPADGELK |
|  |  |  | ALGVEMDLSDKPMGLGVR |
|  |  |  | VLNLEEGGAFTTSSAEEMLK |
|  |  |  | ESLGLGDADVLLLSDGNLELTR |
| 62 | Salt stress root protein RS1 | gi|115435500 | IDFPGAKPVSDAVAK |
|  |  |  | SGTTPLSPAIAFILEK |
|  |  |  | EEPKPEPEAEAAAETTSR |
| 63 | Ribulose-1,5-bisphosphate carboxylase/oxygenase large | gi|109156602 | DTDILAAFR |
|  |  |  | LTYYTPEYETK |
|  |  |  | TFQGPPHGIQVER |
|  |  |  | LTYYTPEYETKDTDILAAFR |
| 64 | Peroxidase | gi|20286 | MGNISPLTGTQGQIR |
|  |  |  | NFASNAAAFSSAFTTAMVK |
|  |  |  | GLLHSDQVLFNGGSADNTVR |
|  |  |  | KGLDATDMVALSGAHTIGQAQCQNFR |
|  |  |  | LHFHDCFVQGCDASVLLSGQEQNAGPNVGSLR |
| 65 | Glyceraldehyde-3-phosphate dehydrogenase A | gi|115458768 | KTLAEEVNQAFR |
|  |  |  | VVDLADIVANQWK |
|  |  |  | GTMTTTHSYTGDQR |
|  |  |  | GDSSPLDVIAINDTGGVK |
|  |  |  | VIAWYDNEWGYSQR |
|  |  |  | YDSTLGIFDADVKPVGDNAISVDGK |
| 66 | Putative chloroplast phosphoglycerate kinase | gi|46981258 | ELDYLVGAVSSPK |
|  |  |  | SVGDLTAADLEGKR |
|  |  |  | FLKPSVAGFLLQK |
|  |  |  | GVSLLLPSDVIIADK |
|  |  |  | LVSELPNGSVLLLENVR |
|  |  |  | LASLADLYVNDAFGTAHR |
|  |  |  | ADLNVPLDDNQNITDDTR |
|  |  |  | KLASLADLYVNDAFGTAHR |
| 67 | Protein disulfide isomerases | gi|7209794 | SVYYGAAEEFKDK |
|  |  |  | AHVEPDQIVSWLK |
|  |  |  | LFKPFDELLVDSK |
|  |  |  | FLIGDLEASQGAFQYFGLR |
| 68 | ATP synthase CF1 beta subunit | gi|50233978 | AHGGVSVFGGVGER |
|  |  |  | FVQAGSEVSALLGR |
|  |  |  | VGLTALTMAEYFR |
|  |  |  | VALVYGQMNEPPGAR |
|  |  |  | QINVTCEVQQLLGNNR |
|  |  |  | DVNKQDVLLFIDNIFR |
|  |  |  | GMEVIDTGAPLSVPVGGATLGR |
|  |  |  | IFNVLGEPVDNLGPVDTSATFPIHR |
| 69 | Salt stress root protein RS1 | gi|115435500 | SGTTPLSPAIAFILEK |
|  |  |  | VAPFVPKEEPKPEPEAEAAAETTSR |
| 70 | 4-nitrophenylphosphatase | gi|115459134 | LIDGVPETLDMLR |
|  |  |  | ELELAGFQYLGGPSDGDK |
|  |  |  | ELELAGFQYLGGPSDGDKK |
|  |  |  | DAVTHLTDAQEWAGGGSMVGAILGSTK |
|  |  |  | IELKPGFYMEHDKDVGAVVVGFDR |
| 71 | ATP synthase CF1 beta subunit | gi|50233978 | AHGGVSVFGGVGER |
|  |  |  | IVGNEHYETAQR |
|  |  |  | FVQAGSEVSALLGR |
|  |  |  | VGLTALTMAEYFR |
|  |  |  | QDVLLFIDNIFR |
|  |  |  | VALVYGQMNEPPGAR |
|  |  |  | QINVTCEVQQLLGNNR |
|  |  |  | DVNKQDVLLFIDNIFR |
|  |  |  | GIYPAVDPLDSTSTMLQPR |
|  |  |  | GMEVIDTGAPLSVPVGGATLGR |
|  |  |  | IFNVLGEPVDNLGPVDTSATFPIHR |
| 72 | ATP synthase gamma chain | gi|115472339 | GLCGSFNNNVLK |
|  |  |  | ALQESLASELAAR |
|  |  |  | QLGLEYTVVSVGK |
|  |  |  | SDPIIQTLLPMSPK |
